# Carnio-malacia, or Softening of the Bones of the Head

**Published:** 1845-09

**Authors:** 


*Cranio-malaciu, or Softening of the Bones of the Head.*—About three years ago, Dr. Widtmann was called to a child, nine months old, who had died suddenly and most unexpectedly on its mother's lap. The attendants drew his attention to the state of the occiput: it was quite devoid of all hair, had a bluish aspect, and was so soft and unresisting to the pressure of the finger, that it yielded like a piece of thin pasteboard. Dr. W. attributing the death to *Laryngeal Asthma*, did not think much of the case, till about a twelve-month afterwards, when he happened to meet with, in the writings of M. Elsaesser on *Atrophy of the cranium*, an exact description of its leading features. According to this author's observations, the occiptal bone, in this morbid state, will often be found on dissection to exhibit several solutions of its osseous continuity, the vacant spaces being then filled up with nothing but membrane. The periosteum is usually highly vascular, thick, and firmly adherent. Such a state of the cranial bones M. Elsaesser regards as the commencement of rickets; this disease subsequently affecting other bones of the skeleton, if the patient survives. As a matter of course, the children affected with it are always poor weak ailing creatures, with big heads, pale bloodless complexions, and tumid bellies. These are the usual victims of that disease which, under the names of *thymic asthma*, *laryngismus stridulus*, *laryngeal asthma*, *&c.*, has attracted so much notice of late years. M. Elsaesser prefers to call it *tetanus apnoieus* (*i. e.* non respirabilis) *periodicus*, and attributes it to a transitory congestion of the brain, which has become the more readily excited in consequence of the attenuation of the cranial bones. The disease most frequently occurs in the second trimestrial period of life. A common symptom in such children is, that they are fretful and uneasy, whenever they are laid down; while they may generally be pacified by taking them up in the arms, and keeping the head well supported.

Dr. Widtmann relates no fewer than *nine* cases of this infantile asthma, in every one of which, he assures us, there was a very obvious and easily recognisable softening of the occipital bone. In one, which proved fatal, this bone is described as being externally of a deep blue colour, and as thin and yielding as a piece of parchment; the diploe was soft and full of a sanguinolent fluid.—*Ibid, from Medicinisches Correspondenzblatt Bayerischer Aertz.*

